# *Sargassum horneri (Turner) C. Agardh* Extract Regulates Neuroinflammation In Vitro and In Vivo

**DOI:** 10.3390/cimb44110367

**Published:** 2022-11-03

**Authors:** Jun Hwi Cho, Dae Hyun Kim, Jong Suk Lee, Mi-Suk Seo, Mi Eun Kim, Jun Sik Lee

**Affiliations:** 1 Department of Life Science, Immunology Research Lab, BK21-Plus Research Team for Bioactive Control Technology, College of Natural Sciences, Chosun University, Dong-gu, Gwangju 61452, Korea; 2 Biocenter, Gyeonggido Business & Science Accelerator (GBSA), Suwon 16229, Gyeonggi-do, Korea; 3 LKBio Inc., Chosun University Business Incubator (CUBI) Building, Dong-gu, Gwangju 61452, Korea

**Keywords:** *S. horneri* extract, microglia, neuroinflammation, MAPK, cytokines

## Abstract

Previously, we reported that *Sargassum horneri (Turner) C. Agardh* (*S. horneri*) is a brown algae species that exerts anti-inflammatory activity toward murine macrophages. However, the anti-neuroinflammatory effects and the mechanism of *S. horneri* on microglia cells are still unknown. We investigated the anti-neuroinflammatory effects of *S. horneri* extract on microglia in vitro and in vivo. In the present study, we found that *S. horneri* was not cytotoxic to BV-2 microglia cells and it significantly decreased lipopolysaccharide (LPS)-induced NO production. Moreover, *S. horneri* also diminished the protein expression of iNOS, COX-2, and cytokine production, including IL-1β, TNF-α, and IL-6, on LPS-stimulated microglia activation. *S. horneri* elicited anti-neuroinflammatory effects by inhibiting phosphorylation of p38 MAPK and NF-κB. In addition, *S. horneri* inhibited astrocytes and microglia activation in LPS-challenged mice brain. Therefore, these results suggested that *S. horneri* exerted anti-neuroinflammatory effects on LPS-stimulated microglia cell activation by inhibiting neuroinflammatory factors and NF-κB signaling.

## 1. Introduction

Neuroinflammation is an essential event to enable recovery from innate immune responses following events, such as nervous tissue damage or microbial infection. It starts with signals, such as brain damage [1]. In neuroinflammation, it plays an important role in cells or molecules and many molecules, such as microglia, pattern-recognition receptors and cytokines [2,3]. Microglia secrete anti-inflammatory factors or mediators that affect BBB integrity [4]. When microglial cells are continuously activated, immune cells become concentrated in the brain [5]. Microglial cells are the representative immune cells of the central nervous system [6] and the major effector cells that mediate neuroinflammation. They are responsible for phagocytosis in the brain where they account for 10% to 15% of brain cells. Activated microglia secrete inflammatory cytokines in several neuroinflammatory diseases [7]. They can actively change their morphology by reacting to infection conditions or signals. Microglia maintain small size in their resting inactive state, but morphology changes in response to damage or pathogens and they migrate through chemotaxis to become phagocytic cells [8]. Activated microglia phagocytose apoptotic cells by releasing pro-inflammatory and other molecules [9]. Neurons that have undergone apoptosis secrete various factors, such as soluble factors and extracellular membrane proteins, which, in turn, induce microglial activity. This process is known as the self-propelling cycle [10]. Microglial BV-2 cells involved in the inflammatory response stimulate Toll-like receptor-4 in response to lipopolysaccharide (LPS) and activate mitogen-activated protein kinases (MAPKs) and nuclear factor-κB (NF-κB) [11]. Once activated, these signaling pathways produce pro-inflammatory cytokines, such as interleukin (IL)-6 and tumor necrosis factor (TNF)-α, and stimulate the expression of enzymes, including inducible nitric oxide synthase (iNOS) and cyclooxygenase (COX)-2, to produce a variety of inflammatory molecules, such as nitric oxide (NO) [12]. Although these inflammatory mediators are needed to remove viruses and tumors, their overproduction can cause serious damage to tissues and lead to chronic inflammatory diseases [11]. Inhibiting inflammatory mediator production may be one way to combat excessive inflammation.

*Sargassum horneri (Turner) C. Agardh* (*S. horneri*) is a brown algae, found in coastal areas of Asia, such as Korea, China and Japan, that has various effects in the body [13,14]. Studies suggest that *S. horneri* can help prevent osteoporosis and control cholesterol, blood pressure and hyperlipidemia [15,16]. Cosmetics and functional foods containing *S. horneri* are known to have anti-aging, anti-allergic and whitening effects [17,18]. We previously reported the anti-inflammatory effects of ethanolic extract from *S. horneri* on LPS-induced macrophage activation [14]. However, no study has assessed its effects on neuroinflammation mediated by microglia. Therefore, we investigated the anti-neuroinflammatory activity of *S. horneri* extract in LPS-induced neuroinflammation.

## 2. Materials and Methods

### 2.1. Reagents

The compound Griess reagent, 3 3-(4,5-Dimethyl-2-thiazolyl)-2,5-diphenyl-2H-tetrazolium bromide (MTT), TRIzol Reagent were purchased from Sigma Chemical (St. Louis, MO, USA). The antibody Anti-p-p65, anti-p65, anti-i-NOS, anti-COX-2, anti-p-p38, anti-p-ERK, anti-p-JNK, anti-JNK were purchased Santacruz (Santa, CA, USA) and anti-ERK and anti-p38 were purchased from Cell signaling (Cell signaling, Danvers, MA, USA).

### 2.2. Cell Culture and Extraction of Sample

BV-2 cells were cultured at 37 °C in the presence of 5% CO_2_ in Dulbecco’s Modified Eagle’s Medium (DMEM) supplemented with 10% FBS, 200 IU/mL penicillin, 200 µg/mL streptomycin. *S. horneri* extract was reconstituted in ethanol and then diluted to the desired concentration in DMEM (final ethanol concentration 0.3%). We collected *S. horneri* in Wando, South Korea, during February 2016. After removing salt and debris, the seaweed was incubated at 37 °C to eliminate moisture. The collected samples were washed gently with clean water three times and then dried with hot air (40 °C) for 2 days. The extract was isolated with 10 volumes (*v/w*) of 70% ethanol at room temperature overnight; this procedure was repeated three times. The extracts were filtered through Whatman filter paper No. 2 (Whatman Ltd., Maidstone, Kent, England), concentrated with a vacuum evaporator and completely dried with a freeze dryer.

### 2.3. UPLC-HR-MS/MS Analysis

Ultra-high-performance liquid chromatography (UPLC) high-resolution tandem mass spectrometry (HR-MS/MS) analysis were carried out using an Orbitrap Fusion (Thermo Electron Co., Waltham, MA, USA) coupled to an Acquity UPLC system (Waters, Milford, MA, USA). Chromatographic separation of ethanol extract was conducted using a Acquity UPLC^®^ BEH C18 column (“2.1 × 150 mm”, 1.7 μm), operated at 50 °C and using mobile phases A (water with 0.1% formic acid) and B (acetonitrile with 0.1% formic acid). The flow rate and injection volumes were 0.4 mL/min and 3 µL, respectively. The elution gradients were as follows: 0–1 min, 5% B (isocratic); 1–20 min, 5–70% B (linear gradient); 20–24 min, 70–100% B (linear gradient); 24–26 min, 100% B (isocratic). The LC-MS system consisted of heated electrospray ionization (HESI) probe as the ionization source. MS analysis was performed with negative ion mode and the following parameters for MS/MS scan: *m*/*z* range of 100–1800; collision-induced dissociation energy of 45%; data-dependent scan mode. The Orbitrap analyzer was used for high-resolution mass spectra data acquisition with a mass resolving power of 60,000 full width at half maximum (FWHM).

### 2.4. Cell Viability Assay

Cell viability was measured by Annexin V/PI staining. The BV-2 cells (2 × 10^6^ cells/well) were seeded in 6-well culture plates with DMEM. Cells were treated with the various concentrations *S. honeri* extract (0~300 µg/mL) and incubated in 37 °C for 24 h. After 24 h, the cells were detached and centrifuged at 20 °C and 2000 rpm for 3 min. We washed 2~3 times with warm PBS, staining using Annexin V/PI Kit and measured using FACS (fluorescence-activated cell sorter).

### 2.5. NO Assay

BV-2 cells (3 × 10^4^ cells/well) were seeded in a 96-well culture plate in DMEM. Cultured cells were pretreated with various concentrations of *S. honeri* extract (0 to 300 µg/mL) for 2 h and then cells were incubated for 22 h in absence or presence of LPS (200 ng/mL). After incubation, the cultured medium was mixed with an equivalent volume of Griess Reagent and incubated for 15 min at room temperature. After 15 min incubation, absorbance was measured using an ELISA microplate reader at 540 nm of absorbance.

### 2.6. Enzyme-Linked Immunosorbent Assay (ELISA)

BV-2 cells (3 × 10^4^ cells/well) were seeded in 96-well culture plate. Cells were pretreated with various concentrations of *S. honeri* extract (0 to 300 µg/mL) for 2 h and then cells were incubated for 22 h in absence or presence of LPS (200 ng/mL). After incubation, supernatant was used for samples and the quantification of TNF-α, IL-1β and IL-6 release was measured by Mouse IL-1β, IL-6 and TNF-α ELISA MAX Deluxe Sets (BioLegend, CA, USA), according to manufacturer’s protocol. Briefly, standards and samples were incubated for 1 h and Avidin-HRP bind to detection antibody. For visualization, substrate solution was added to each well and then the reaction was stopped by stop solution (2 N H_2_SO_4_). Absorbance was measured by ELISA microplate reader at 405 nm wavelength.

### 2.7. Western Blot Analysis

BV-2 cells (2 × 10^6^ cells/dish) were seeded in 60 mm culture dish in DMEM. Cultured cells starved 4 h in serum-free media DMEM and exposed to LPS (200 ng/mL) in the absence or presence of 300 µg/mL of *S. honeri* extract pretreatment. Following 15, 30 and 45 min of incubation at 37 °C, cells were washed twice with cold PBS and lysed with modified RIPA buffer containing (150 mM sodium chloride, 1% Triton X-100, 0.5% sodium deoxycholate, 0.1% sodium dodecyl sulfate (SDS), 50 mM Tris (pH 8.0), 1 mM phenylmethylsulfonyl fluoride (PMSF), 2 g/mL leupeptin, 1 µg/mL pepstatin, 1 mM sodium orthovanadate and 100 mM sodium fluoride) for 30 min at 4 °C. Lysates were cleared by centrifuging at 14,000× *g* for 15 min at 4 °C. The protein content of cell lysates was determined using the Micro BCA assay kit (Pierce, Rockford, IL, USA).

Equivalent amounts of proteins were separated by 10 or 12% SDS-polyacrylamide gel electrophoresis (SDS-PAGE) and electrophoretically transferred to a polyvinylidene difluoride (PVDF) transfer membrane. The membrane was placed into a blocking solution (5% skim milk and 2% BSA) at room temperature for 1 h. After blocking, anti-ERK1/2, anti-phospho-ERK1/2 (p-ERK), anti-p38, anti-phospho-p38 (p-p38), anti-NF-κB (NF-κB-p65) and anti-phospho-NF-κB-p65 antibodies overnight (Santa Cruze Biotechnology, Santa Cruz, CA, USA) were used as the primary antibodies. Horseradish peroxidase-conjugated anti-rabbit and anti-mouse antibodies (Santa Cruze Biotechnology) were used as the secondary antibodies. Band detection was performed using the enhanced chemiluminescence (ECL) detection system and exposed to radiographic film. Pre-stained blue markers were used for molecular-weight determination.

### 2.8. In Vivo Experiment

All experiments were approved and performed in accordance with the regulations of the Chosun University Care and Use Committee (IACUC, 201901). Mice were housed in a temperature-controlled (21–23 °C) room under a 12:12 h light/dark cycle and provided food and water. C57/BL6 (8 weeks) male mice were randomly divided into 3 groups of five mice per group (Control, LPS, LPS + *S. honeri*). *S. honeri* was dissolved in sterile saline and pre-administered by intraperitoneal injection (i.p.) (10 and 20 mg/kg) for 3 days prior to LPS injection. Further, LPS group was injected intraperitoneally (1 mg/kg) [19,20] for 24 h. Mice brain samples were analyzed after mice were scarified via CO_2_ euthanasia.

### 2.9. Immunohistochemistry

The mice were intra-cardinally perfused with 10 mM phosphate-buffered saline (PBS) and, subsequently, 4% paraformaldehyde. The brain was extracted and post-fixed in 4% paraformaldehyde for 24 h at 4 °C. Fixed brain was transferred to 30% sucrose solution. These brain samples were embedded in OCT for frozen sections and then coronally sectioned at 40 μm using a freezing microtome (MICROM, Walldorf, Germany). Brain sections were washed and blocked with blocking solution for 30 min at room temperature. The sections were incubated with anti-ionized calcium-binding adapter molecule 1 (Iba-1, microglia maker) antibody and glial fibrillary acidic protein (GFAP, astrocytes maker) (Wako Chemical USA, Inc., Richmond, VA, USA) in TBS-TS at 4% overnight. Brain sections were washed with TBS and incubated with anti-mouse IgG labeled with Alexa Fluor 488 and 568 for 3 h at room temperature, respectively. Confocal fluorescence images were acquired FV10i fluoview confocal microscope (Olympus, Tokyo, Japan). The images were analyzed using ImageJ software (NIH, Bethesda, MD, USA).

### 2.10. Statistical Analysis

The results are presented as the mean ± standard deviation. The data were analyzed via analysis of variance (ANOVA) followed by Scheffe’s post hoc test using SPSS. The differences were considered statistically significant at *p* < 0.05.

## 3. Results

### 3.1. S. horneri Extract Inhibits NO Production in LPS-Stimulated BV-2 Microglial Cells

**Figure 1:**
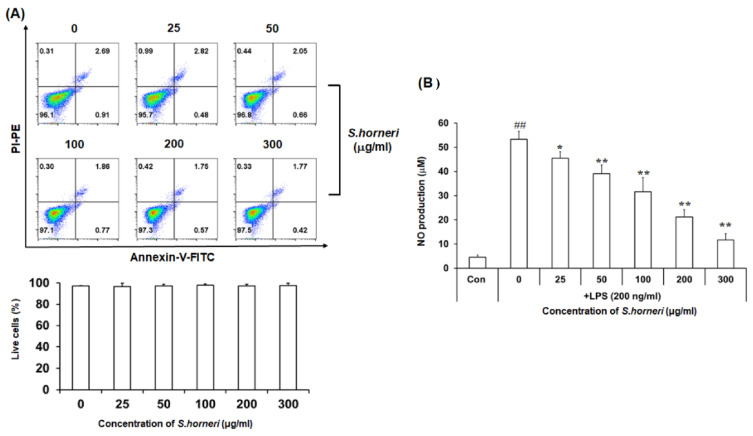
Anti-neuroinflammatory effect of *S. horneri* extract on NO production in LPS-stimulated BV-2 microglial cells. Microglial cell viability was determined by Annexin-V/PI assay after *S. horneri* extract treatment with BV-2 microglial cells (**A**). After *S. horneri* extract treatment of BV-2 microglial cells stimulated with LPS (200 ng/mL), NO production ability was determined by Griess assay (**B**). The result is representative of three repeated independent experiments. Experimental results were indicated as mean (±SE). * *p* <0.005, ** *p* < 0.001, significantly different from LPS-treated group; ## *p* < 0.001, significantly different from LPS-untreated group.

We first investigated the effect of *S. horneri* extract on cell viability and NO regulation. Annexin V/PI staining showed that *S. horneri* extract was not cytotoxic at doses up to 300 µg/mL (Figure 1A). To determine the effect of *S. horneri* extract on NO production, BV-2 microglial cells were pretreated with *S. horneri* extract for 2 h and then stimulated with LPS (200 ng/mL). NO production was increased in LPS compared to control, but NO levels decreased in a dose-dependent manner with *S. horneri* extract treatment (0–300 µg/mL) (Figure 1B). These results indicate that *S. horneri* effectively inhibits NO production in LPS-stimulated BV-2 microglia.

### 3.2. S. horneri Extract Reduced LPS-Induced iNOS and COX-2 Protein Expression

**Figure 2:**
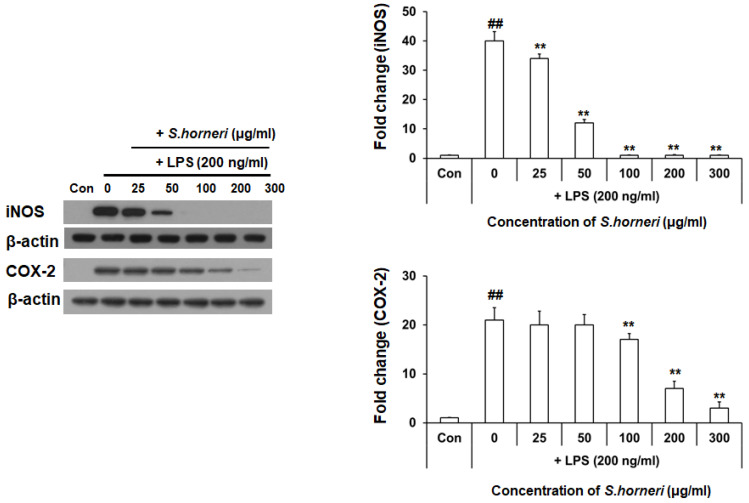
Effect of *S. horneri* extract on protein expression of iNOS and COX-2 in LPS-stimulated BV-2 microglial cells. *S. honeri* extract was pretreated with BV-2 microglial cells 2 h and then incubated with 200 ng/mL LPS for 22 h. iNOS and COX-2 protein expression was determined by Western blot. The result is representative of three repeated independent experiments. Experimental results were indicated as mean (±SE). ** *p* < 0.001, significantly different from LPS-treated group; ## *p* < 0.001, significantly different from LPS-untreated group.

Since *S. horneri* extract inhibits NO production, we assessed its impact on protein expression of iNOS, which is a pro-inflammatory enzyme producing NO and cyclooxygenase (COX)-2. Western blot analysis was performed to confirm that *S. horneri* extract inhibits the expression of pro-inflammatory enzymes. BV-2 microglial cells were pretreated for 2 h with *S. horneri* extract and then stimulated with LPS (200 ng/mL) for 22 h. As shown in Figure 2, protein levels of iNOS and COX-2 were also decreased in BV-2 microglial cells pretreated *S. honeri* extract (0–300 µg/mL) for 2 h, even though expression of both proteins was stimulated by LPS treatment. These results indicate that *S. horneri* extract inhibits protein expression of iNOS and COX-2 in LPS-stimulated BV-2 microglial cell.

### 3.3. S. horneri Extract Reduced Neuroinflammatory Factors in LPS-Stimulated BV-2 Microglial Cells

**Figure 3:**
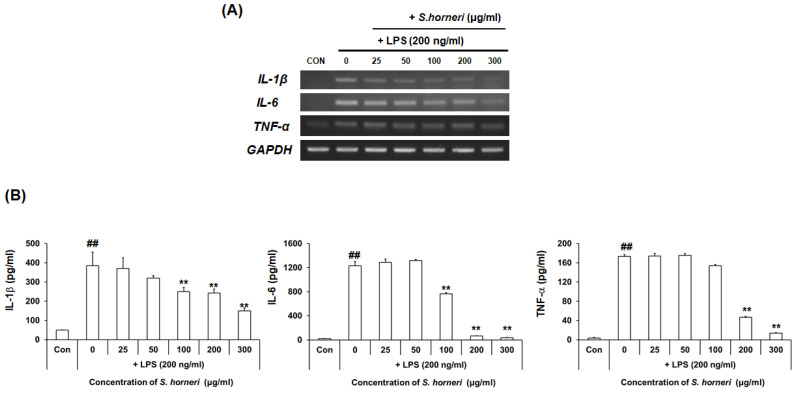
Effect of *S. horneri* extract on the production levels of IL-1β, IL-6 and TNF-α in LPS-stimulated BV-2 microglial cells. *S. horneri* extract was pretreated with BV-2 microglial cells 2 h and then incubated with 200 ng/mL LPS for 6 h. RNA was isolated and analyzed by RT-PCR. IL-1β, TNF- α and IL-6 gene expression was confirmed, respectively (**A**). The cells were pre-treated with *S. horneri* extract (25, 50, 100, 200 and 300 μg/mL) for 2 h, followed by treatment of LPS (200 ng/mL) for 24 h; the levels in the supernatants were measured by ELISA (**B**). Each bar indicates the mean ± SE of four independent experiments. ** *p* < 0.001, significantly different from LPS-treated group; ## *p* < 0.001, significantly different from LPS-untreated group.

We next used RT-PCR to measure mRNA levels of the pro-inflammatory substances IL-6, TNF-α and IL-1β and determined whether their expressions were inhibited by *S. horneri* extract. BV-2 microglial cells were pretreated for 2 h with *S. horneri* extract and then stimulated with LPS (200 ng/mL) for 6 h. As shown in Figure 3A, expression of pro-inflammatory genes, such as IL-6, TNF-α and IL-1β, was increased in LPS-stimulated BV-2 microglial cells, but *S. honeri* extract dose-dependently decreased mRNA expression.

To determine whether levels of IL-6, TNF-α and IL-1β secreted by BV-2 microglial cells were decreased by *S. horneri* extract, IL-6, TNF-α and IL-1 are pro-inflammatory cytokines expressed by BV-2 microglial cells following exposure to LPS. The results confirmed that IL-6 and TNF-α produced from LPS-stimulated BV-2 microglial cells were significantly decreased by pretreatment with *S. horneri* extract. We, therefore, suggest that IL-1β, IL-6 and TNF-α production can be inhibited by *S. horneri* extract, suggesting that the compound has anti-neuroinflammatory effects.

### 3.4. S. horneri Extract Inhibited Phosphorylation of p38 MAPK, ERK and NF-κB

**Figure 4:**
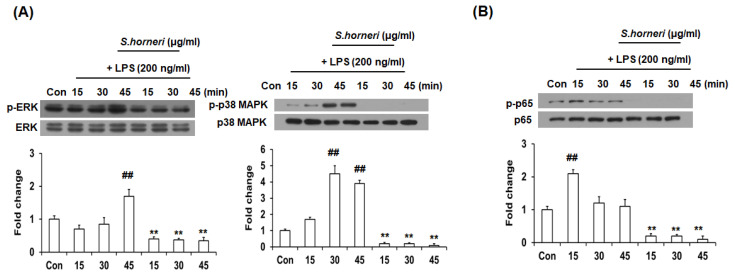
*S. horneri* attenuated LPS-induced MAPK phosphorylation and NF-κB translocation. Cells were pre-treated with 300 μg/mL of *S. honeri* for 2 h, followed by treatment of LPS (200 ng/mL) for 15, 30 and 45 min. The expression of phosphorylated proteins was measured by Western blot. The total extracts and nucleus extracts were assayed with antibodies specific for p-ERK, p-38 MAPK and p-p65 (NF-κB). Total form and β-actin were used as internal control for total fraction. The blots are representative of three independent experiments (**A**,**B**). Each bar indicates the mean ± SE of four independent experiments. ** *p* < 0.001, significantly different from LPS-treated group; ## *p* < 0.001, significantly different from LPS-untreated group.

MAPKs, such as p38 MAPK, JNK and ERK, play important roles in regulating cell growth, division, stress and cytokine-mediated cellular responses; they are involved in signal pathways that regulate inflammatory mediators through transcription factor activation. To clarify how *S. horneri* inhibits inflammatory mediators, we confirmed that it inhibited LPS-induced MAPK activation signaling pathways. p38 MAPK and ERK phosphorylation was increased in BV-2 microglial cells stimulated by LPS for 15, 30 or 45 min, but this was inhibited by *S. honeri* extract treatment. Interestingly, p-p38 MAPK phosphorylation was significantly decreased by *S. horneri* extract treatment (Figure 4A). NF-κB is a transcription factor that regulates the intracellular synthesis of various molecules, including the expression of inflammatory cytokines and iNOS. Therefore, we asked whether the anti-neuroinflammatory activity of *S. honeri* extract was due to inhibited NF-κB activity. As shown in Figure 4B, phosphorylation of p65 was increased in the nucleus of LPS-stimulated BV-2 cells after 15, 30 and 45 min, but p65 phosphorylation was decreased in the nucleus by *S. horneri* extract treatment. This suggests that *S. horneri* extract strongly inhibited NF-κB migration to the nucleus upon activation. Collectively, these results indicate that the inhibitory effect of the *S. horneri* extract on pro-inflammatory cytokine production in LPS-induced BV-2 cells is mediated by p38 MAPK, ERK and NF-κB signaling.

### 3.5. S. horneri Extract Attenuates Astrocyte and Microglia Activation in the Mice Brain

**Figure 5:**
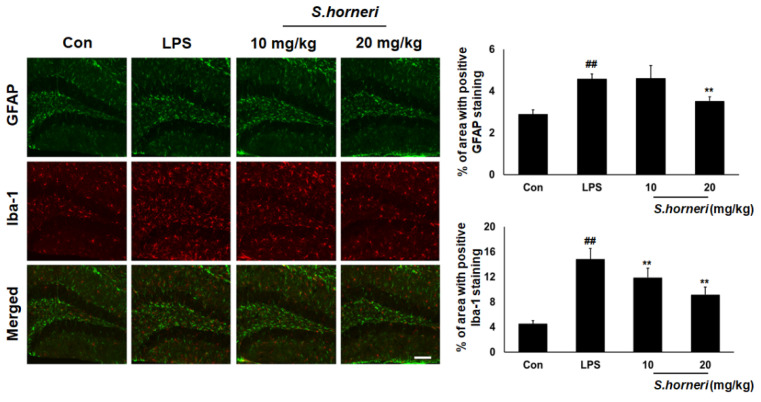
*S. horneri* inhibited LPS-induced astrocyte and microglia activation in brain hippocampus. C57BL/6 mice were randomly divided into four groups. The control group was challenged with the same amount of solvent i.p. (*n* = 5). The treatment group was administered LPS (1 mg/kg) and *S. honeri* (10 and 20 mg/kg), following in vivo study design. Brain sections were immunostained with anti-GFAP (Green) or anti-Iba-1 (Red) antibody as described in the Materials and Methods section (scale bar = 100 μm). The result is representative of three repeated independent experiments. Each bar indicates the mean ± SE of four independent experiments. ** *p* < 0.01, significantly different from LPS-treated group; ## *p* < 0.01, significantly different from LPS-untreated group.

To confirm anti-neuroinflammatory activity of *S. horneri* in brain-resident glial cells, LPS-induced astrocyte and microglia activation were examined in mouse brain. GFAP (astrocyte marker) and Iba-1 (microglia marker) were highly expressed in the hippocampus in LPS-injected groups compared with control groups. As shown in Figure 5, LPS-induced astrocyte activation in the hippocampus was significantly decreased by 20 mg/kg of *S. horneri* administration. Moreover, LPS-induced microglia activation in the hippocampus was significantly decreased by *S. horneri* administration in a dose-dependent manner. These data suggested that *S. horneri* attenuates the LPS-induced neuroinflammatory response by inhibiting astrocyte and microglia activation.

### 3.6. Identification of S. horneri Extract Using LC-MS/MS Analysis

**Figure 6:**
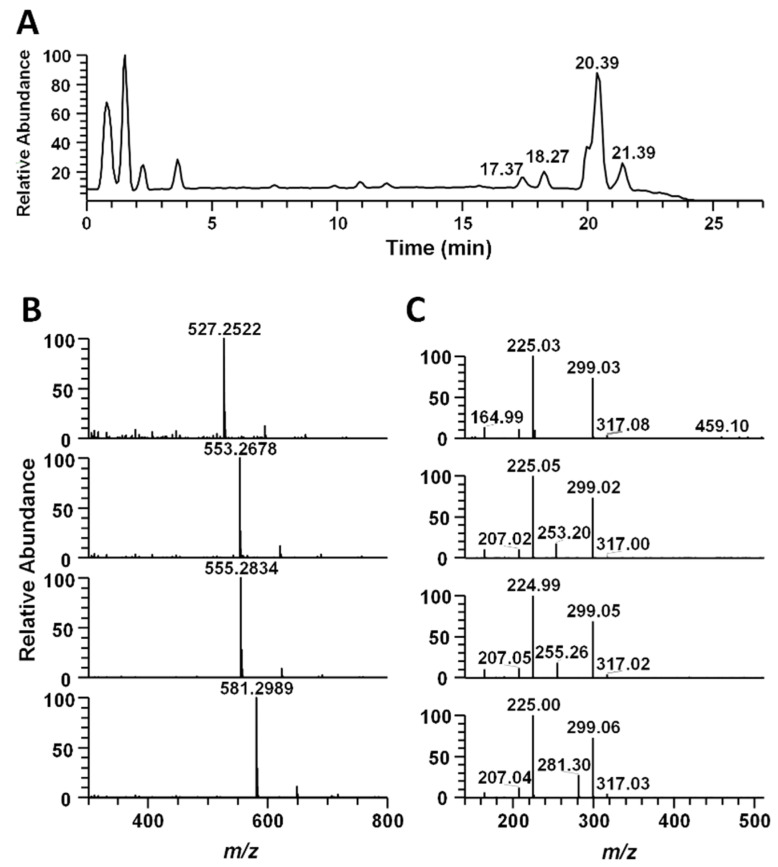
The high-resolution UPLC-MS/MS result of ethanol extract of marine brown alga *S. horneri*. (**A**) Mass chromatogram of the ethanol extract at negative ion mode. (**B**) High-resolution mass spectrum of 4 sulfoglycolipid compounds at retention times 17.37, 18.27, 20.39 and 21.39 min. (**C**) Tandem mass spectrum of each of the 4 sulfoglycolipid compounds (*m*/*z* 527.2522, 553.2678, 555.5834 and 581.2989).

In order to confirm the active ingredient in *S. horneri*, we performed LC-MS/MS analysis. The main peak was identified as 1-O-Hexadecanoyl-3-O-(6′-sulfo-a-D-quinovopyranosyl) glycerol by AntiBase database (John Wiley & Sons, Inc. Chichester, England) search and previously reported data by Z. Cui et al. [21]. The other peaks were predicted as related isomer compounds by tandem mass analysis. (Figure 6). The high-resolution mass spectrum showed an *m*/*z* 527.2522, 553.2678, 555.2834 and 581.2989 ([M-H]), which was tentatively identified as 1-O-Tetradecanoyl-3-O-(6′-sulfo-a-D-quinovopyranosyl) glycerol, 1-O-(11-Hexadecenoyl)-3-O-(6′-sulfo-a-D-quinovopyranosyl) glycerol, 1-O-Hexadecanoyl-3-O-(6′-sulfo-a-D-quinovopyranosyl) glycerol, 1-O-(9-Octadecenoyl)-3-O-(6′-sulfo-a-D-quinovopyranosyl) glycerol based on the high-resolution mass and AntiBase search (Table 1). Furthermore, the structural correlation of the four peaks was confirmed through MS/MS spectrum analysis.

## 4. Discussion

*S. horneri* is a seaweed that exerts various effects on the body. Previous studies reported that *S. horneri* can prevent osteoporosis and help control cholesterol, blood pressure and hyperlipidemia [13,14,17]. However, no mechanism has been described for how it could regulate microglia activity. In the present study, we investigated the anti-neuroinflammatory effect of *S. horneri* on LPS-stimulated microglia activation.

NO is a highly reactive factor involved in vasorelaxation, neurotransmission and cell-mediated immune responses [22,23,24]. NO production mediates the inflammatory response and exerts cytotoxic activity against external pathogens. LPS-induced BV-2 microglial cells act as inflammatory mediators downstream of NO to induce pro-inflammatory cytokines, such as IL-6, IL-1β and TNF-α; they also enhance inflammation by secreting COX-2 [14,25]. Moreover, NO overexpression aggravates various inflammatory diseases. Therefore, substances that inhibit inflammatory mediators that produce NO and pro-inflammatory cytokines may be useful for treating various immune and inflammatory diseases. We found that *S. honeri* treatment significantly inhibited NO production and iNOS expression on LPS-stimulated microglial cells in a dose-dependent manner (Figure 1B and Figure 2). These results indicated the effect that *S. horneri* could suppress neuroinflammation because it reduced the production of NO.

In previous studies, activated microglia induced the production of neuroinflammatory factors, such as IL-1β, IL-6 and TNF-α, and can induce neuroinflammatory responses [25]. Therefore, we investigated the amount of inflammatory factors produced by the *S. honeri* treatment. As shown in Figure 3, gene expression and production levels of IL-1β, IL-6 and TNF-α were increased by LPS treatment, but decreased in a concentration-dependent manner by *S. horneri* treatment in LPS-stimulated BV2 microglial cells. These results confirmed the possibility of developing a neuroinflammation agent, as *S. horneri* inhibits the production of NO and inflammatory factors important for neuroinflammation.

In order to identify the factors related to the regulation of neuroinflammation by *S. horneri*, we next tried to identify the signaling pathway proteins related to regulation of neuroinflammation. Among the signaling proteins involved in the regulation of neuroinflammatory factors, MAPK is highly related. MAPKs include JNK, ERK and p38 MAPK and play important roles in various cellular processes, such as cell differentiation, proliferation and inflammation [26]. Inactive MAPK remains in the cytosol and is activated by phosphorylation in response to LPS or other stimuli, at which point it translocates to the nucleus and stimulates cytokine production [19]. In this study, *S. horneri* pretreatment reduced phosphorylated ERK and p38 MAPK in LPS-stimulated BV-2 microglial cell activation (Figure 4A). Therefore, we found that *S. horneri* inhibits neuroinflammation by regulating ERK and p38 MAPK.

NF-κB plays important roles in inflammatory reactions and in various immune responses, tumorigenesis and autoimmune diseases [27,28,29]. NF-κB is present in the cytoplasm in an inactive state bound to Iκ-Bα, but in response to external stimuli, such as LPS, Iκ-Bα is phosphorylated and the separated NF-κB translocates to the nucleus where it is involved in the production of NO and pro-inflammatory cytokines [30]. As shown in Figure 4B, we found that NF-κB migration into the nucleus was strongly decreased following pretreatment with *S. horneri*. These results demonstrated that *S. horneri* suppressed the expression of NO and neuroinflammatory factors by regulating MAPK and NF-κB signaling pathways. Moreover, we performed LC-MS/MS analysis to find out the major components in *S. horneri’s* inhibitory effect on neuroinflammation and identified four active compounds (Figure 6 and Table 1). Although four compounds were identified through LC-MS/MS analysis, additional studies on their anti-neuroinflammatory effects should be conducted.

Microglia and astrocyte activation is important in neuroinflammatory responses. It also induces various CNS injuries, such as Alzheimer’s and Parkinson’s disease [25]. Therefore, in order to determine the inhibitory effect of *S. horneri* on neuroinflammation, we analyzed microglia and astrocyte activation by *S. horneri* administration in LPS-challenged mice. As shown in Figure 5, we found that microglia and astrocyte were activated in the group treated with LPS. However, microglia and astrocyte activation were significantly inhibited in the 20 mg/kg *S. horneri* pretreatment group. This result suggests that *S. horneri* has a preventive effect on neuroinflammation in an animal model.

## 5. Conclusions

Our results show that *S. horneri* attenuated neuroinflammation via the activation of MAPK and NF-κB pathways, including ERK and p38 MAPK, in LPS-stimulated BV-2 cells, thereby inhibiting the production of NO and the pro-inflammatory cytokines IL-6 and TNF-α. These findings indicate that *S. horneri* may be an effective treatment for neuroinflammatory diseases.

## Figures and Tables

**Table 1:** Metabolite profiling of crude ethanol extract of *S. horneri*.

RT (min)	*m*/*z* ([M-H])	Formula	Δppm	Compound Identification
17.37	527.2522	C23H43O11S	−1.775	1-O-Tetradecanoyl-3-O-(6′-sulfo-a-D-quinovopyranosyl) glycerol
18.27	553.2678	C25H45O11S	−1.782	1-O-(11-Hexadecenoyl)-3-O-(6′-sulfo-a-D-quinovopyranosyl) glycerol
20.39	555.2834	C25H47O11S	−1.938	1-O-Hexadecanoyl-3-O-(6′-sulfo-a-D-quinovopyranosyl) glycerol
21.39	581.2989	C27H49O11S	−2.092	1-O-(9-Octadecenoyl)-3-O-(6′-sulfo-a-D-quinovopyranosyl) glycerol

## Data Availability

The data presented in this study are available on request from the corresponding authors.
